# Editorial: Clinical metagenomics-based diagnostics for infectious diseases

**DOI:** 10.3389/fcimb.2024.1459621

**Published:** 2024-08-07

**Authors:** Jeyaprakash Rajendhran, Pushpanathan Muthuirulan, Arun Prasath Lakshmanan, Sathyavathi Sundararaju

**Affiliations:** ^1^ Department of Genetics, School of Biological Sciences, Madurai Kamaraj University, Madurai, India; ^2^ Department of Human Evolutionary Biology, Harvard University, Cambridge, MA, United States; ^3^ Precision Nutrition, Research Department, Sidra Medicine, Doha, Qatar; ^4^ Department of Pathology, Sidra Medicine, Doha, Qatar

**Keywords:** clinical metagenomics, next-generation sequencing (NGS), metagenomic NGS (mNGS), infectious diseases, diagnosis, pathogen detection

Infectious diseases remain a significant global public health challenge, leading to over 13 million deaths annually (Cohen, 2000). The development of rapid and dependable diagnostic tools for infectious diseases is crucial. Traditionally, the diagnosis of infectious diseases typically involves culture-based methods, serological tests, or PCR assays. However, these methods have limitations, including time consumption, lack of specificity, and unculturability of certain organisms. Metagenomic approaches, particularly clinical metagenomics using metagenomic next-generation sequencing (mNGS) technology, offer a promising solution (Batool and Galloway-Peña, 2023). This emerging method, with its potential to significantly reduce the global burden of morbidity and mortality, instills hope and optimism in our fight against infectious diseases.

Clinical mNGS is revolutionizing diagnostics, particularly in intensive care units (ICUs), where rapid and precise pathogen identification is critical (Liang et al., 2023). It can identify a wide range of pathogens, including bacteria, viruses, fungi, and parasites, and is adept at detecting co-infections and delineating complex microbial ecosystems often missed by conventional methods. This comprehensive diagnostic capability of mNGS provides reassurance in the face of complex infectious diseases. The application of mNGS, with its role in promoting targeted therapeutic interventions, empowers healthcare professionals and significantly enhances therapeutic decision-making and optimizing antibiotic use. Importantly, deploying mNGS in clinical settings supports antimicrobial stewardship by helping to avoid the overuse of broad-spectrum antibiotics and promoting the use of more appropriate, targeted therapies.

Clinical mNGS uses NGS to analyze genetic material from clinical samples, providing a comprehensive snapshot of the microbial communities. mNGS produces a large amount of data, which can be challenging to interpret and definitively identify the causative agent. Standard operating procedures for sample processing, sequencing, quality control, and subsequent bioinformatic analyses must be established to make mNGS suitable for regular clinical use. This Research Topic included articles covering advances from sample processing to data analysis and clinical applications of mNGS in diagnosing and treating infectious diseases.

He et al. presented a bibliometric analysis using the Web of Science on the application of mNGS in pathogen diagnosis. Their analysis revealed that 325 mNGS studies were published between 2009 and 2022. The most studied infections included pneumonia, tuberculosis, central nervous system infections, and infections in children. The number of publications on mNGS has been increasing every year, indicating the expansion of research in this field.

Du et al. have reported a case study where a 30-year-old woman was initially misdiagnosed with tuberculosis. The anti-tuberculosis treatment did not work, and eventually, she developed sepsis. Subsequently, through mNGS, she was diagnosed with talaromycosis caused by *Talaromyces marneffei*. After receiving appropriate antifungal treatment, her health condition improved. Xu et al. reported another case study where *T. marneffei* infection was diagnosed in a renal transplant patient. They detected *T. marneffei* sequences in blood and bronchoalveolar lavage fluid using mNGS and then confirmed it with a blood culture. This study showed the value of using mNGS for diagnosis and highlighted the importance of starting antifungal treatment promptly, which helped the patient recover successfully.

Wu et al. have reported that mNGS was used to diagnose pneumonia caused by *Chlamydia psittaci*. Four patients were admitted with pneumonia symptoms, and their bronchoalveolar lavage fluid (BALF) samples underwent multiple tests, such as acid-fast bacteria, fungal, galactomannans tests, and tumor marker tests, and found all the tests were negative. However, chest CT scans revealed multiple infectious lesions in both lungs. Also, serum inflammatory markers like C-reactive protein (CRP) were elevated in all patients. Subsequently, mNGS testing was performed on BALF samples obtained through bronchoscopy, which revealed *Chlamydia psittaci* infection. As a result of the diagnosis, the antibiotic treatments were modified, leading to the successful treatment of the patients. Xie et al. investigated the microbiomes in the lower respiratory tracts of patients with chlamydial pneumonia using mNGS. They found that patients infected with *C. psittaci* had more co-infecting pathogens than those infected with *Chlamydia abortus*. Furthermore, they observed that different clinical subgroups exhibited significantly distinct profiles of lower respiratory tract microbiomes. Mixed infections involving *C. psittaci* and *C. abortus* were linked to lower lung microbiome diversity, indicating that chlamydial infections shape the unique lung microbiome and pathology. Wu et al. also reported that mNGS significantly improved the accuracy and detection rate of pathogens in patients with pulmonary infections after examining more than 500 BALF samples.

Chen et al. analyzed sputum samples from 50 patients with pulmonary infections following cardiac surgery using conventional culture testing and mNGS. They identified 64 bacterial pathogens, ten fungal pathogens, and three viruses. Importantly, they distinguished between opportunistic pathogenic and colonizing strains in the individual samples. These findings offer valuable insights into the precise diagnosis of pathogens, particularly opportunistic pathogens, in a clinical setting.

Zhao et al. conducted a study comparing a new mNGS tool called Quality/Quantity mNGS (QmNGS) with the standard mNGS for diagnosing pulmonary pathogens. They evaluated the bronchoalveolar lavage fluid from 36 patients with suspected pulmonary infection using both UmNGS and QmNGS. The study found that the sensitivity of QmNGS was similar to that of mNGS, while the specificity of QmNGS was slightly higher. However, it was noted that the depth and coverage of the QmNGS sequencing were lower than those of UmNGS. Chen et al. introduced a 16S rDNA nanopore sequencing method called NB16S-seq by adding pathogen-specific barcodes to the 16S rRNA gene primers. This improved mNGS tool enabled the rapid identification of common pulmonary bacterial pathogens in bronchoalveolar lavage fluid samples from children with severe pneumonia.

Zhang et al. assessed the clinical advantages of mNGS in hematological patients with and without hematopoietic stem cell transplantation (HSCT). They found that mNGS is highly sensitive in detecting pathogens and can be a basis for anti-infective therapies in hematological diseases. However, they noted that mNGS cannot wholly replace traditional detection methods. They suggested that mNGS of peripheral blood can serve as a valuable complementary detection method when traditional tests are negative or when specimens are challenging to obtain. In a study, Liu et al. assessed the diagnostic accuracy and clinical significance of blood mNGS in ICU patients suspected of having mono- and polymicrobial bloodstream infections. The research revealed that mNGS demonstrated better diagnostic accuracy and higher sensitivity than blood culture, particularly for polymicrobial infections. Consequently, mNGS has the potential to play a crucial role in accurately diagnosing and treating infections in critically ill patients. Cao et al. analyzed circulating cell-free DNA using mNGS from blood samples of ICU patients at high risk of bloodstream infections. They aimed to determine if a modified mNGS (mcfDNA-seq) version could detect pathogens before blood cultures indicated any positive infection. The results showed a higher diagnostic and overall predictive sensitivity of mcfDNA-seq, suggesting its potential use in identifying pathogens before the onset of bloodstream infections.

The application of mNGS of urine samples has been reported by Huang et al. for diagnosing urinary tract infections (UTIs) among patients undergoing cutaneous ureterostomy (CU). The study found that mNGS could efficiently detect pathogens and assist in the early diagnosis of UTI in CU patients, making it helpful for monitoring microbial changes in their urine. Additionally, mNGS could detect the presence of genes coding for virulence factors and antimicrobial resistance, facilitating appropriate antimicrobial therapy. It’s essential to ensure that metagenomic DNA extraction is reliable and reproducible so that mNGS can be used effectively for pathogen detection. Zhang et al. compared three DNA extraction methods for urine samples with microbial infections to address this. They used long-read nanopore sequencing to evaluate metagenomic DNA yield, integrity, and microbial diversity. Based on their findings, they recommend using an enzyme-based method for metagenomic DNA extraction from urine for mNGS-based pathogen detection.

Zhang et al. evaluated the use of mNGS in detecting pathogens responsible for spinal infections. They used the infected tissue and pus samples for mNGS and culturing. Pathogenic organisms like *Mycobacterium tuberculosis* complex, *Staphylococcus aureus*, *Mycoplasma hominis*, and *Brucella* spp. were identified in multiple samples through mNGS analysis. The results emphasized its superior sensitivity and specificity compared to microbial culture, reaffirming mNGS’s importance in guiding treatment decisions and enhancing patient outcomes. Xu et al. also reported the superiority of mNGS over culture methods in detecting pathogens responsible for spinal infections using blood and tissue samples. Notably, novel pathogens, including non-tuberculosis mycobacteria, other fungi, and bacterial species, were detected using mNGS. Sepsis is a leading cause of death in patients with cervical spine injury (CSI). Wan et al. conducted a study to assess the effectiveness of mNGS in identifying pathogens in CSI patients with sepsis. They tested 27 blood samples from 17 patients using mNGS and found that it could detect a wide range of pathogens, including 129 bacterial species, eight viral species, and 51 fungal species. The authors concluded that while mNGS does not have prognostic value, it can help guide antibiotic therapy in CSI patients diagnosed with sepsis.

Central nervous system (CNS) infections can be fatal and require rapid medical intervention. However, accurate detection of pathogens in cerebrospinal fluid (CSF) samples has been challenging due to the small sample volumes and low detection efficiency of traditional culture methods. Yu et al. evaluated the effectiveness of mNGS in diagnosing CNS infections using CSF samples from 390 patients. They analyzed both cell-free and whole-cell DNA from the CSF and found that mNGS outperformed traditional methods in detecting pathogens, especially in viral and mycobacterial CNS infections. Zhang et al. also performed mNGS of CSF samples from patients infected with community-acquired central nervous system infections (CA-CNS). The study identified various pathogens responsible for CA-CNS infections, many of which could not be detected by conventional methods.

Cancer patients, particularly those with weakened immune systems, are at high risk of developing infections. Having reliable and fast diagnostic methods for identifying infections in these patients is crucial. In a study by Deng et al., the effectiveness of nanopore amplicon sequencing in detecting pathogens in immunocompromised cancer patients with suspected infections was evaluated. The researchers tested samples of BALF, blood, sputum, urine, and peritoneal fluid from the suspected patients. It is a modified version of mNGS, where PCR-amplified products are sequenced with barcodes. The results demonstrated that nanopore amplicon sequencing enables early detection of infections and facilitates precise treatment with anti-infective medications. Colorectal cancer (CRC) is one of the most prevalent cancers. Following surgery to resect the primary tumor in CRC patients, stoma construction is typically performed. Sakai et al. compared the microbiome of CRC patients with and without a stoma using fecal mNGS. Their findings revealed a reduction in anaerobic microbial composition in patients with a stoma. Additionally, they observed an underrepresentation of genes associated with methane and short-chain fatty acid production in stoma patients.

Preeclampsia (PE) is a pregnancy complication with severe hypertension and multiple organ damage. In a study by Lv et al., the fecal samples from 40 early-onset PE patients and 37 healthy pregnant women were analyzed using mNGS. The study found that the presence of certain species, such as *Blautia*, *Pauljensenia*, *Ruminococcus*, and *Collinsella*, as well as specific microbial functions, were associated with PE. Zhang et al. assessed the diagnostic accuracy of mNGS, Xpert, and droplet digital PCR (ddPCR) in detecting Mycobacterium tuberculosis (MTB) in various clinical samples such as BALF, pleural effusion, pericardial effusion, and ascites. Their findings revealed that ddPCR exhibited the highest sensitivity compared to mNGS and Xpert for diagnosing active MTB cases. In a study by Huang et al., fungal co-infections in critically ill patients during the Omicron variant outbreak of COVID-19 from December 2022 to January 2023 were investigated using BALF and blood mNGS. The study revealed that mNGS detected a significantly higher number of pathogenic microorganisms than traditional methods, especially in detecting fungi and viruses. Aspergillus infection was the most common; most patients had concurrent bacterial or viral infections. The study emphasized the superior detection rates of mNGS compared to conventional methods and highlighted the importance of early intervention for fungal infections in COVID-19 patients.

The use of clinical mNGS for diagnostics is a groundbreaking advancement in the field of infectious disease. Articles on this Research Topic discuss using mNGS to identify bacterial, fungal, and viral infections in the blood, BALF, CSF, sputum, peritoneal fluid, urine, and fecal samples. mNGS can also be used to detect microbiome dysbiosis. There is potential to expand the use of mNGS to diagnose any infectious disease from any biological sample. However, it is crucial to establish a standard protocol for diagnosing various diseases using mNGS. mNGS offers comprehensive, rapid, and accurate pathogen identification, potentially significantly improving patient outcomes, mainly when traditional methods are insufficient. While challenges remain, ongoing innovation and collaboration among the scientific and medical communities will enhance the robustness and feasibility of mNGS in combating infectious diseases.

## Author contributions

JR: Writing – original draft, Writing – review & editing. PM: Writing – original draft, Writing – review & editing. AL: Writing – original draft, Writing – review & editing. SS: Writing – original draft, Writing – review & editing.
